# Supplementary material for: Development of the 12-Base Short Dimeric Myogenetic Oligodeoxynucleotide That Induces Myogenic Differentiation
Source: BioTech (Basel). 2024 Apr 25;13(2):11. doi: 10.3390/biotech13020011 (PMC11130974; doi:10.3390/biotech13020011)
Supplement: Supplementary file 1 [file biotech-13-00011-s001.zip › biotech-2943914-supplementary.pdf]

BioTech

MDPI

Supplementary Materials: Development of the 12-Base Short Dimeric Myogenetic Oligodeoxynucleotide That Induces Myogenic Differentiation

Koji Umezawa, Rena Ikeda, Taiichi Sakamoto, Yuya Enomoto, Yuma Nihashi, Sayaka Shinji, Takeshi Shimosato, Hiroshi Kagami and Tomohide Takaya

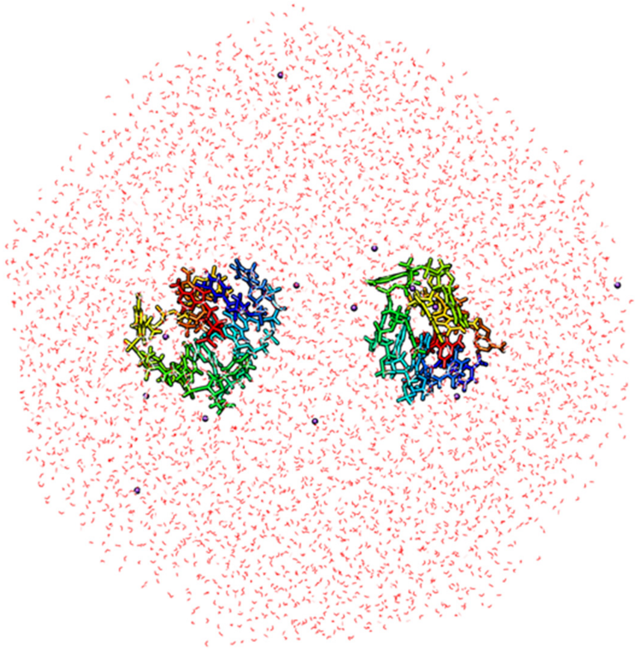

Supplementary Figure S1. The simulation system of the iMyo01-DNA dimer. iMyo01-DNA molecules are represented as a rainbow-colored stick model from the 5′ to the 3′ end. The solvent molecules are shown as red wires (oxygen atoms of water molecules), purple spheres (K^+ ions), and a green sphere (Cl^- ion).

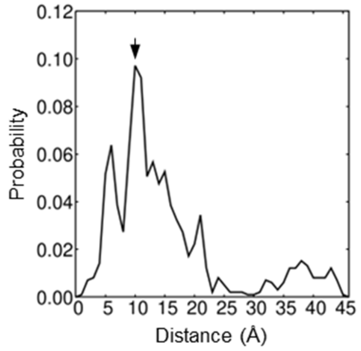

Supplementary Figure S2. The probability of the center-of-mass distances between the two iMyo01-DNA molecules. The McMD simulation at 310 K provided 988 conformations as a canonical ensemble. Their center-of-mass distances and probability distribution are shown. The dimer state defined below 25 Å of the distance contains 855 conformations (86.5%), and the separated monomer state contains 133 conformations (13.5%). An arrow indicate the peak at d_{com} ≈ 10 Å.

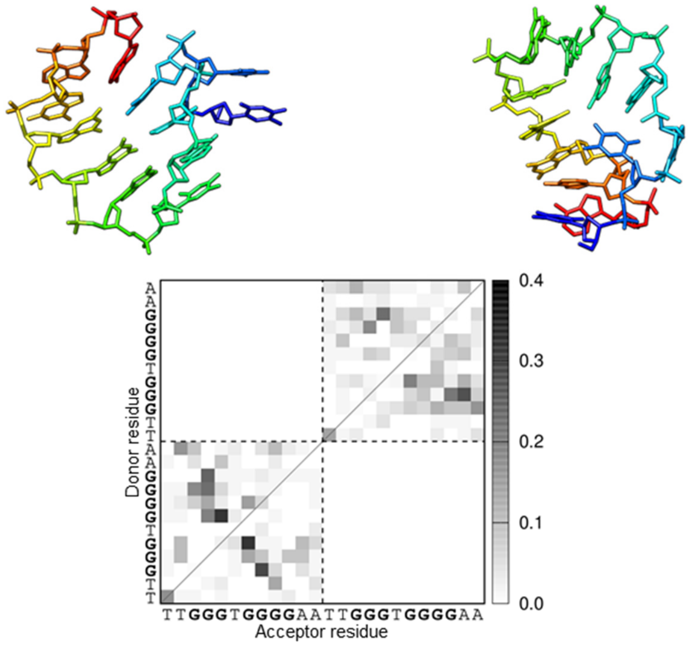

Supplementary Figure S3. The representative iMyo01-DNA structure in the separated monomer state. The structures (d_{com}, 37.8 Å) are shown as a rainbow-colored stick model from the 5′ to the 3′ end. The hydrogen bond pattern is plotted as a monochrome map. The gray scale indicates the probability of hydrogen bond formation between residues. The two iMyo01-DNA sequences are displayed sequentially on the axes.
